# Imaging Monitoring of Kupffer Cell Function and Hepatic Oxygen Saturation in Preneoplastic Changes During Cholangiocarcinogenesis

**DOI:** 10.1038/s41598-017-14218-x

**Published:** 2017-10-27

**Authors:** Seunghyun Lee, Jung Hoon Kim, Jeong Hwa Lee, Yoh Zen, Joon Koo Han

**Affiliations:** 1 Department of Radiology, Seoul National University Hospital, Seoul, Korea; 2 Institute of Radiation Medicine, Seoul National University Hospital, Seoul, Korea; 3 Department of Diagnostic Pathology, Kobe University Graduate School of Medicine, Kobe, Japan

## Abstract

We investigated serial changes of the Kupffer cell (KC) function and hepatic oxygen saturation (sO_2_) using contrast-enhanced ultrasound imaging (CEUS) and photoacoustic imaging (PAI) in preneoplastic changes during cholangiocarcinogenesis induced by obstructive cholangitis and N-nitrosodimethylamine in a mouse model. The CEUS and PAI were performed to assess Sonazoid contrast agent uptake by KC and changes in the sO_2_ of liver parenchyma. An extensive bile ductular reaction, cystic dilatation, and epithelial hyperplasia with dysplastic changes were noted in the experimental group. During the preneoplastic changes, the parenchymal echogenicity on the Kupffer-phase of CEUS was continuously decreased in the experimental group, and which means that the Sonazoid phagocytosis by KC was decreased. The number of KCs was increased in the CD68 analysis, indicating functionally impaired KCs. There was a simultaneous serial decrease in sO_2_ on PAI measurement of the experimental group during the preneoplastic changes. The experimental group also showed significantly higher expression of hypoxia-inducible factor-1α and vascular endothelial growth factor protein. Our study demonstrated that KC dysfunction and hypoxic environmental changes were the factors influencing preneoplastic change during cholangiocarcinogenesis, and we could non-invasively monitor these changes using CEUS and PAI.

## Introduction

Cholangiocarcinoma (CCA) is a devastating cancer arising from malignant transformation of cholangiocytes which are the epithelial cells lining biliary epithelium^1^. Although the pathophysiology of CCA is poorly understood, it has been noted that the known risk factors, such as liver fluke infections, chronic hepatitis, cirrhosis, and toxins, share the common feature of including cholestatic injury and/or chronic liver inflammation^1–3^. As a result of chronic biliary epithelial injury or inflammation, biliary epithelium is known to be malignantly transformed through a multistep process with epithelial hyperplasia or dysplasia^4^. The precursor lesions, such as biliary intraepithelial neoplasia or intraductal papillary neoplasm of the bile ducts, result from biliary epithelial-cell hyperplasia leading to dysplasia and eventually adenocarcinoma^5,6^.

Over the last years a number of animal models of CCA have been developed for a better understanding of the pathophysiology^3^. The bile duct ligation (BDL) causes obstructive cholestatic injury associated with chronic biliary epithelial inflammation and altered Kupffer cell (KC) function which is known as the precursor mechanism of carcinogenesis^7^. *N*-nitrosodimethylamine (NDMA) is also one of the carcinogens that promotes activation of KCs and causes the DNA structure damage in mice^3^. Previous studies have noted that it has an important role in the development of preneoplastic lesions in that activated KCs release biologically active products including reactive oxygen species and cytokines^8^. Hypoxia, an important microenvironment feature in chronic inflammatory diseases, also likely contributes to carcinogenesis and angiogenesis in hepatic carcinogenesis^9^.

However, there have only been a few studies regarding the image monitoring of the changes in the tissue microenvironment, including the KC function or tissue oxygen saturation (sO_2_) in the carcinogenesis. Previously published studies have shown that the function of KCs can be evaluated using contrast-enhanced ultrasound (CEUS) with Sonazoid in rat models^10,11^. Newly developed photoacoustic imaging (PAI) is also a real-time noninvasive and quantitative imaging modality for the study of tissue hypoxia without using a radioisotope or contrast agent^12^.

Therefore, our study investigated image monitoring of serial changes of the KC function and hepatic sO_2_ using CEUS with Sonazoid and PAI in preneoplastic changes occurring during cholangiocarcinogenesis induced by obstructive cholangitis and NDMA in a mouse model.

## Results

**Figure 1:**
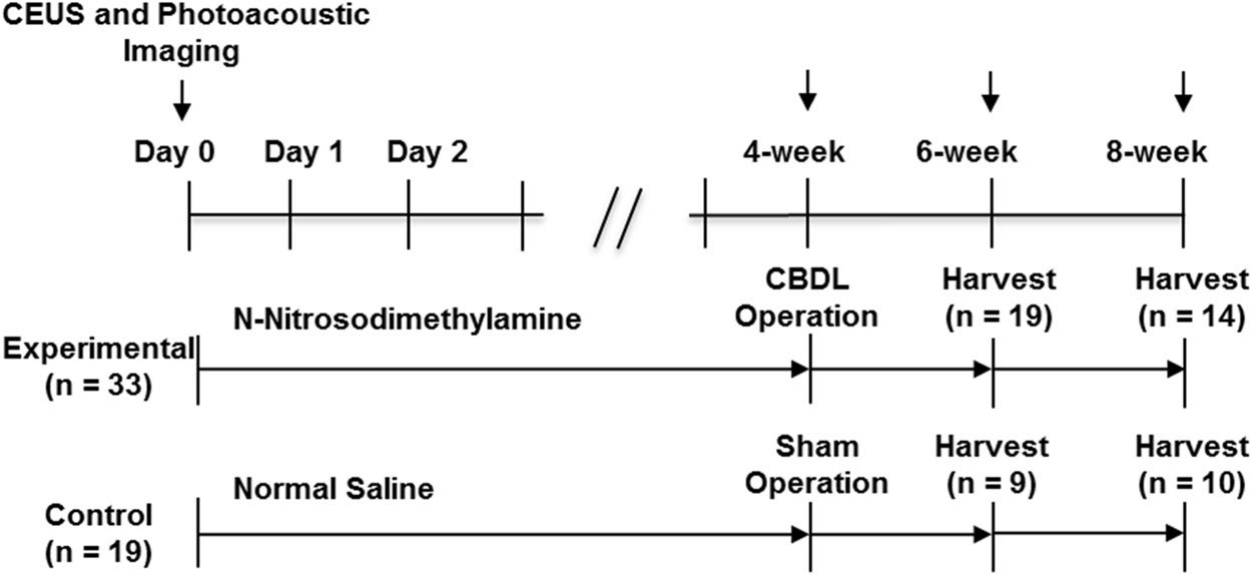
Experimental Design. Fifty-two mice were divided randomly into two groups as follows: 19 mice receiving normal saline, and 33 mice receiving *N*- nitrosodimethylamine (NDMA) in drinking water. Four weeks after NDMA administration, common bile duct ligation (CBDL) was performed in the experimental group, whereas sham operation was performed in the control group. The contrast-enhanced ultrasound (CEUS) and photoacoustic imaging were performed at baseline, four-, six- and eight-week follow-up, and then tumor was harvested at six weeks and eight weeks following administration of NDMA or normal saline.

A total of 52 mice, including the experimental group (n = 33) and the control group (n = 19), could be assessed for Sonazoid uptake and CD68 expression of KCs (Fig. 1). The degree of sO_2_ and expression of hypoxia-inducible factor-1α (HIF-1α) and vascular endothelial growth factor (VEGF) protein in liver parenchyma was also evaluated in each group.

### Preneoplastic Changes during Cholangiocarcinogenesis

**Figure 2:**
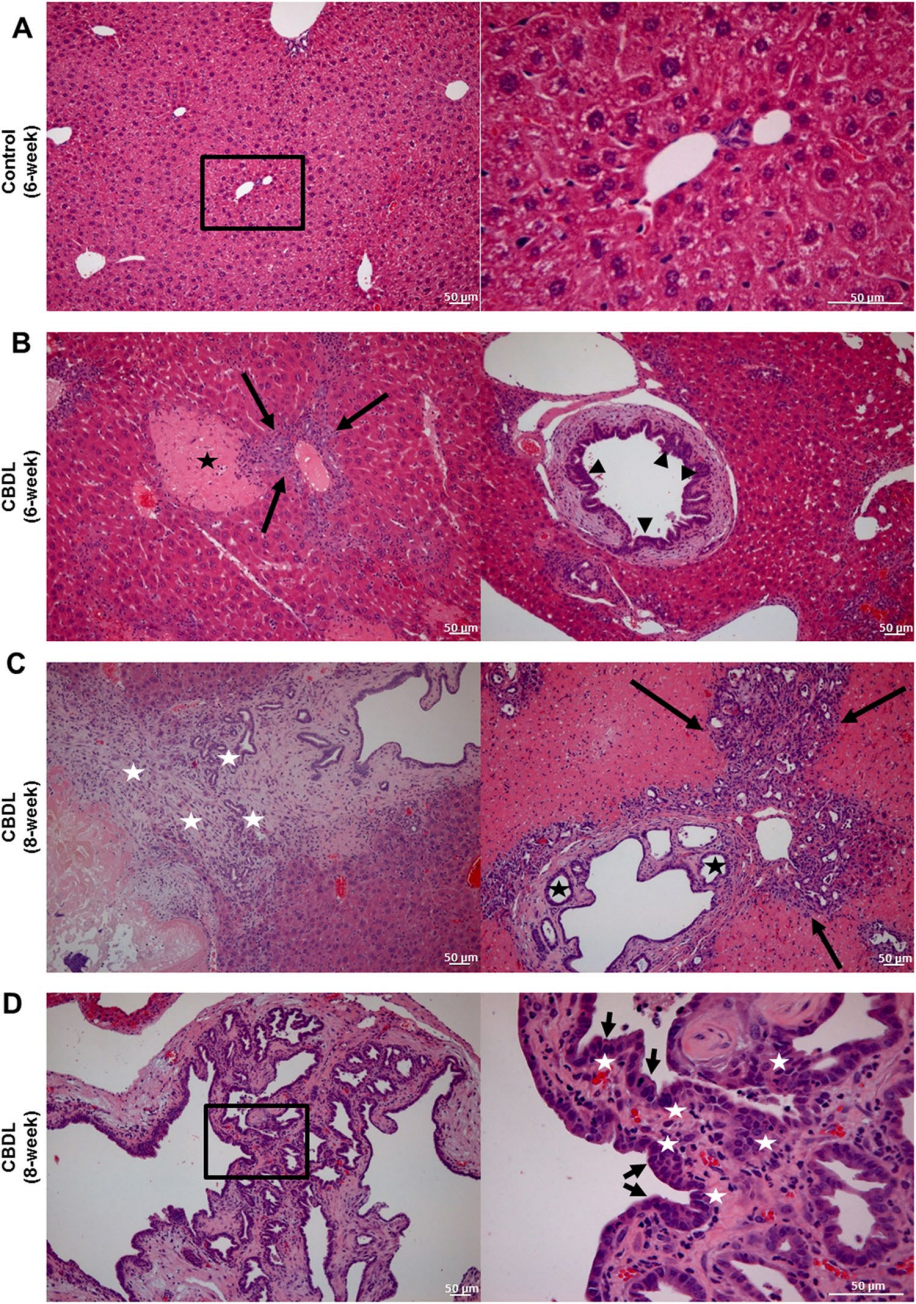
Preneoplastic Changes during Cholangiocarcinogenesis. **(A)** The livers of the control group showed a normal appearance of hepatocytes, cholangiocytes, and portal triad without evidence of inflammation (original magnification; left: ×40 and right: ×200, scale bars = 50 µm). **(B)** The experimental group showed that there was bile ductular reaction with a periductular inflammatory infiltrate (arrows), zonal necrosis (star), and epithelial hyperplasia (arrowheads) at six weeks (original magnification; ×40, scale bars = 50 µm). (**C**) At eight weeks, there was granulation tissue and bile ductular reaction (white stars) adjacent to necrotic area. An extensive bile ductular reaction with a periductular inflammatory infiltrate (arrows), and the cystic dilatation with epithelial hyperplasia (black star) were noted around the small portal tract (original magnification; ×40, scale bars = 50 µm). **(D)** The large bile duct showed that there was biliary epithelial hyperplasia with irregularly shaped ductal architecture (arrow), and the lining epithelium had enlarged nuclei and slightly distorted polarity (white stars) (original magnification; left ×40 and right: ×200, scale bars = 50 µm). These findings was consistent with preneoplastic changes during cholangiocarcinogenesis.

On histopathologic examination, the livers of the control group showed a normal appearance of hepatocytes, cholangiocytes, and portal triad without evidence of inflammation (Fig. 2A). However, the experimental group showed that there was periductal fibrosis and epithelial hyperplasia in the large bile duct and bile ductular reaction with a periductular inflammatory infiltration around small portal tracts at six weeks (Fig. 2B). At eight weeks, there was an extensive bile ductular reaction with periductular inflammatory infiltrates as well as cystic dilatation with epithelial hyperplasia in the small portal tract. Granulation tissue and bile ductular reaction adjacent to necrosis were noted (Fig. 2C). The large bile ducts also showed epithelial hyperplasia with irregular-shaped ductal architecture and cystic dilatation with enlarged nuclei and slightly distorted polarity (Fig. 2D). Although the biliary epithelial lining was clearly hyperplastic with foci of dysplasia, invasive cancers were not identified (Fig. 2D). These changes were already observed at six week and became more prominent at eight weeks. The histologic findings are summarized in Table 1 as classified into inflammatory changes and preneoplastic changes. The inflammatory changes included zonal necrosis, granulation tissue, periductular infiltration, periductal fibrosis, and cystic dilatation. The preneoplastic changes included epithelial hyperplasia and epithelial dysplasia. Both inflammatory and preneoplastic changes became a more common occurrence as time progressed. At 8-week, the experimental group showed the epithelial hyperplasia (14/14, 100%) and dysplasia (12/14, 85.7%), and these findings were considered to be preneoplastic changes (Table 1).

**Table 1:** Preneoplastic Changes during Cholangiocarcinogenesis.

Histologic Findings	Experimental Group (n = 33)	
	6-week (n = 19)	8-week (n = 14)
**Inflammatory Changes**		
Zonal necrosis	11 (57.9%)	10 (71.4%)
Granulation tissue	3 (15.8%)	11 (78.6%)
Periductular infiltration	18 (94.7%)	14 (100.0%)
Periductal fibrosis	15 (78.9%)	14 (100.0%)
Cystic dilatation	10 (52.6%)	9 (64.3%)
**Preneoplastic Changes**		
Epithelial hyperplasia	**16 (84.2%)**	**14 (100.0%)**
Epithelial dysplasia	**3 (15.8%)**	**12 (85.7%)**

Note— The table shows the number and percentage of histologic findings in the experimental group. The control group did not show inflammatory change nor preneoplastic change in histologic findings.

### Changes in Kupffer-phase Echogenicity and the Kupffer Cell Fraction

**Figure 3:**
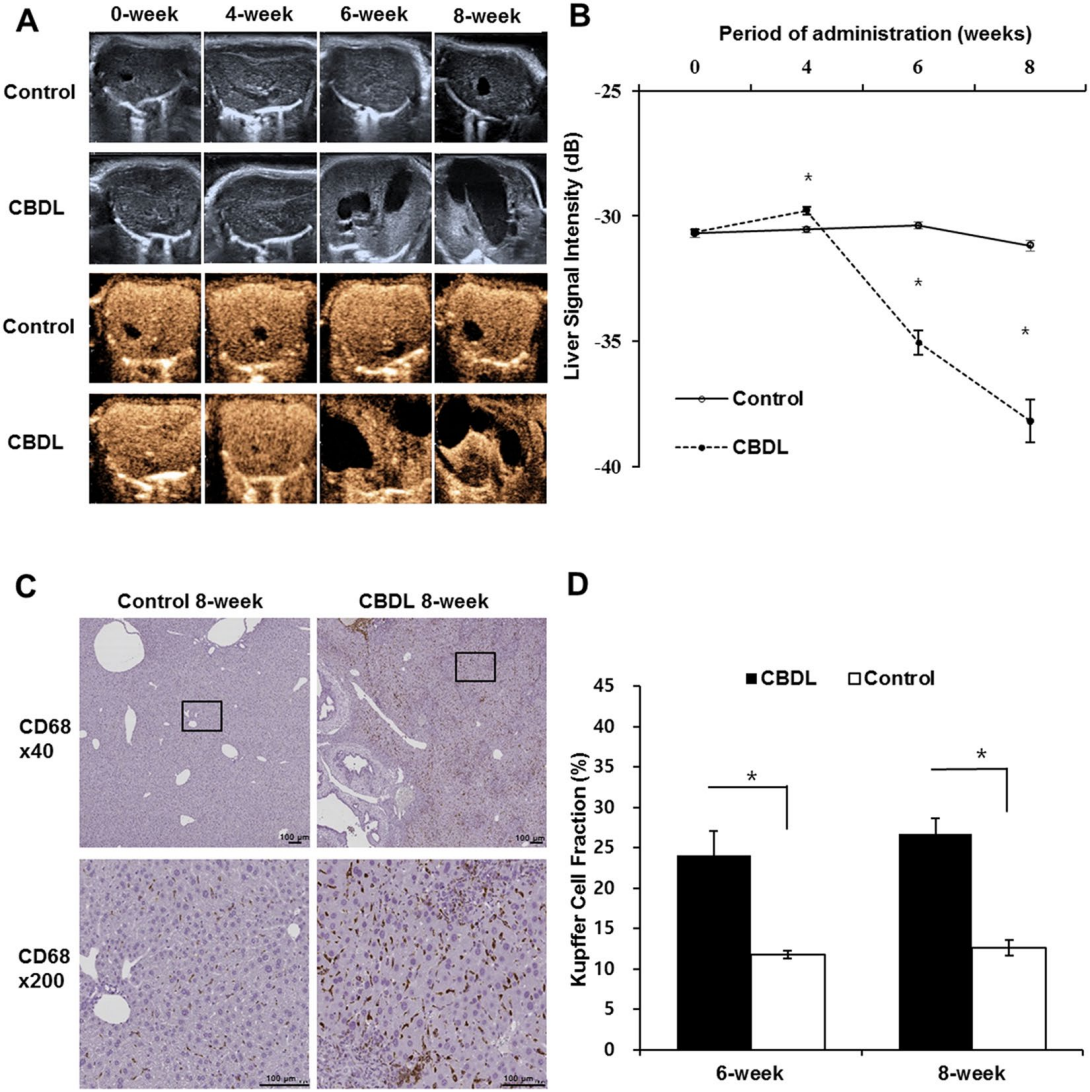
Changes in Kupffer-phase Echogenicity and the Kupffer cell Fraction. **(A)** In the representative case, the bile duct dilatation became increasingly clear from the four-week to eight-week follow-up in the experimental group. The liver parenchymal echogenicity on Kupffer phase by contrast-enhanced ultrasound imaging (CEUS) decreased compared to that of the control group. **(B)** The vertical axis is the signal intensity (dB) and the horizontal axis is the period of *N*-nitrosodimethylamine or normal saline administration. The parenchymal echogenicity in the experimental group at six- and eight-week on the Kupffer phase of CEUS decreased compared to those of the control group (all *P* < 0.001). **(C)** There was significant increase in CD68 count in the experimental group at six- and eight-week specimen (*P* < 0.001) (original magnification; lower power: ×40 and high power: ×200, scale bars = 100 µm). **(D)** The KC fraction of the experimental group tended to increase slightly from six weeks to eight weeks (24.1 ± 4.7 vs. 12.0 ± 2.2% and 27.4 ± 7.8 vs. 12.6 ± 3.6%). *P < 0.05.

The bile duct dilatation became increasingly clear from the four-week to the eight-week follow-up in the experimental group. The baseline parenchymal echogenicity on the Kupffer phase of CEUS did not differ significantly between the experimental group and the control group (−30.6 ± 0.9 dB vs. −30.7 ± 1.0 dB, *P* = 0.752). Four weeks after the baseline imaging, the parenchymal echogenicity in the experimental group was slightly increased compared to that of the control group (−29.8 ± 1.3 dB vs. −30.5 ± 0.7 dB, *P* = 0.001). However, the parenchymal echogenicity in the experimental group seen at the six- and eight–week on the Kupffer phase of CEUS, had decreased compared to that of the control group (−35.1 ± 3.8 dB vs. −30.4 ± 0.9 dB and −38.2 ± 4.4 dB vs. −31.2 ± 1.0 dB, all *P* < 0.001). The difference in the parenchymal echogenicity between the experimental and the control group increased from six to eight weeks. **(**Fig. 3A,B **)**.

The KC fractions in the experimental group according to the CD68 examination were significantly higher than those of the control group at six weeks and eight weeks (24.1 ± 4.7 vs. 12.0 ± 2.2% and 27.4 ± 7.8 vs. 12.6 ± 3.6%, all *P* < 0.001). The KC fraction of the experimental group tended to increase slightly from six weeks to eight weeks (Fig. 3C,D).

### Changes in Hepatic Oxygen Saturation and Hypoxia-related Protein Expression

**Figure 4:**
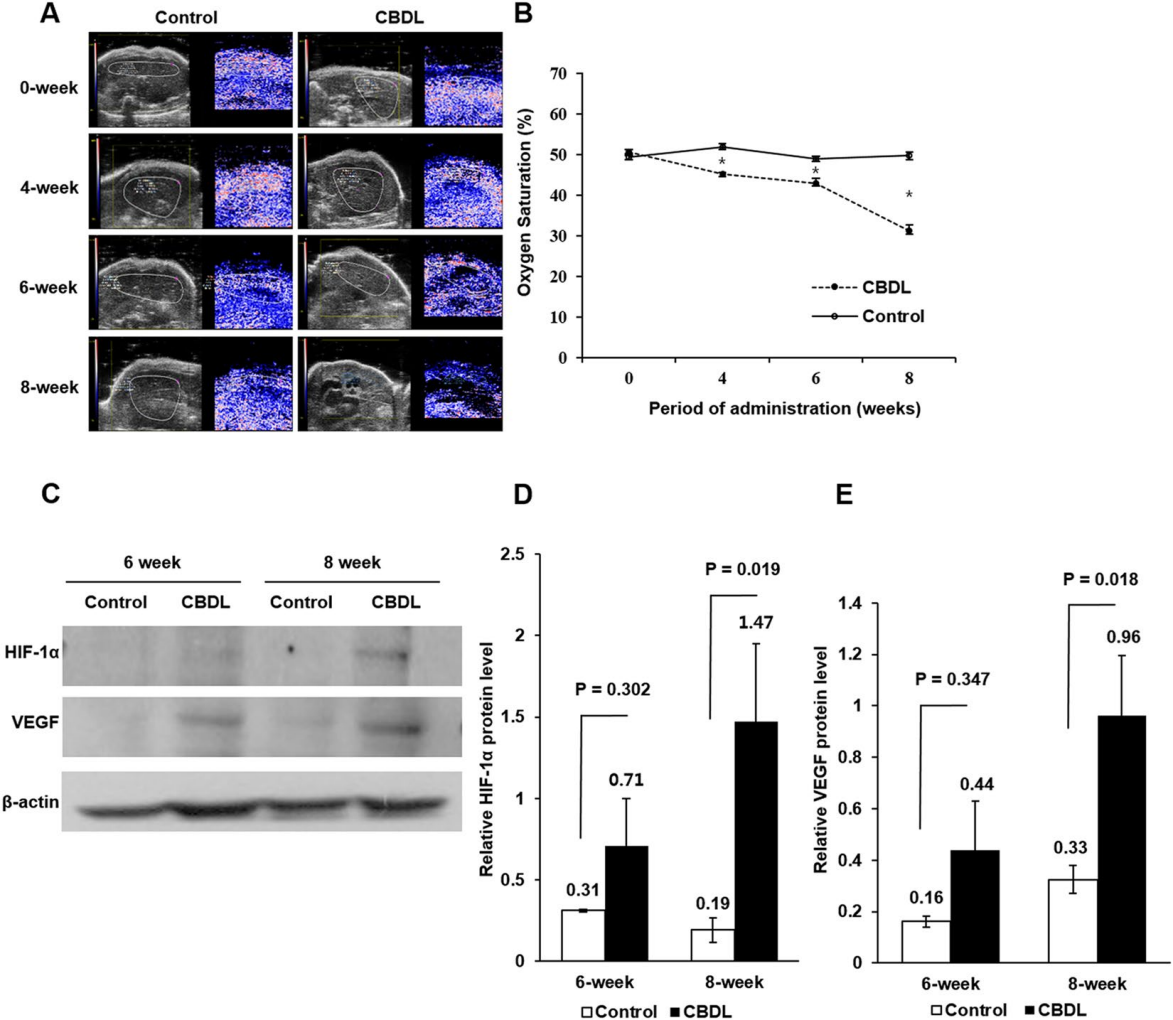
Changes in Hepatic Oxygen Saturation and Hypoxia-related Protein Expression. **(A)** In the representative case, the hepatic oxygen saturation in the experimental group was lower than that in the control group at four, six, and eight weeks (51.6 vs. 54.5%, 48.4 vs. 53.9%, 32.3 vs. 43.2%, and 31.2 vs. 42.9%). **(B)** There was significant decrease of oxygen saturation of liver parenchyma in the experimental group compared to those of control group (45.2 vs. 51.9%, 43.0 vs. 49.0%, and 31.3 vs. 49.7%) at the four, six, and eight weeks, respectively (*P* < 0.001). **(C)** In the Western blot analysis, the experimental group was relatively higher expression of hypoxia-inducible factor-1α (HIF-1α) and vascular endothelial growth factor (VEGF) protein than the control group at eight weeks. The full-length blots with these antibodies were presented in supplementary Figure S1. **(D**,**E)** The experimental group showed significantly higher expression of HIF-1α and VEGF protein than the control group at eight-week (1.47 ± 0.48 vs. 0.19 ± 0.08 and 0.96 ± 0.23 vs. 0.33 ± 0.06, *P* = 0.019 and *P* < 0.018). *P < 0.05.

We did not find a significant difference between the experimental group and the control group in terms of the baseline sO_2_ on PAI (50.6 ± 5.3% vs. 49.4 ± 4.5%, *P* = 0.263). However, the sO_2_ in the experimental group was lower than that in the control group at four, six, and eight weeks (45.2 ± 4.0 vs. 51.9 ± 4.4%, 43.0 ± 10.2 vs. 49.0 ± 3.9%, and 31.3 ± 7.3 vs. 49.7 ± 4.0%, all *P* < 0.001). The difference in the sO_2_ between the experimental group and the control group increased from four to eight weeks (Fig. 4A,B).

To further reveal the induced hypoxic environment during carcinogenesis, we assessed the expression of hypoxia-related protein using Western blot analysis. Even though the experimental group showed higher expression of HIF-1α and VEGF protein than the control group, there was no significant difference at six weeks (0.71 ± 0.29 vs. 0.31 ± 0.01 and 0.44 ± 0.19 vs. 0.16 ± 0.02, *P* = 0.302 and *P* = 0.347). However, the experimental group showed significantly higher expression of HIF-1α and VEGF protein than the control group at eight weeks (1.47 ± 0.48 vs. 0.19 ± 0.08 and 0.96 ± 0.23 vs. 0.33 ± 0.06, *P* = 0.019 and *P* < 0.018) **(**Fig. 4C–E **)**.

## Discussion

Our results showed that KC dysfunction and hypoxic environmental changes were the factors of preneoplastic change during cholangiocarcinogenesis induced by obstructive cholangitis and NDMA in a mouse model, and we could non-invasively monitor these changes using CEUS with Sonazoid and PAI. CEUS with Sonazoid in the experimental group showed that the liver parenchymal echogenicity was reduced, and which indicates that the Sonazoid uptake by KCs was reduced. However, an increase in the number of KC fractions suggested that functionally impaired KCs increased during the preneoplastic change. HIF-1α and VEGF proteins were also expressed in response to hypoxia, while sO_2_ of liver parenchyma decreased on PAI during preneoplastic change. Therefore, we determined that KC dysfunction and hypoxic environmental changes were the factors of preneoplastic change during cholangiocarcinogenesis, and we could non-invasively monitor these changes using CEUS and PAI.

Cholangiocarcinogenesis is a multi-step process dependent on an interaction between genetic and environmental factors^1^. The precursor lesions, such as biliary intraepithelial neoplasia and intraductal papillary neoplasm of the bile ducts, are believed to gradually progress to CCA through molecular changes such as alteration of oncogenes and tumor suppressor genes^5,13^. Chronic biliary tract inflammation is known to progress to hyperplasia or dysplasia of bile epithelium, and which pathways are induced by inflammatory mediators or tissue responses and leading to adenocarcinoma^4^. Our experimental group showed that there were zonal necrosis, granulation tissue, periductular infiltration, periductal fibrosis and cystic dilatation, and these findings were considered to be inflammatory changes. At 8-week, the experimental group showed the epithelial hyperplasia (14/14, 100%) and dysplasia (12/14, 85.7%), and these findings were considered to be preneoplastic changes. Our study did not demonstrate the development of CCA arising from the chronic inflammation and NDMA administration. However, we found biliary epithelial hyperplasia with dysplastic cells, which meant the premalignant condition of CCA such as biliary intraepithelial neoplasia (BilIN), in our animal model.

KCs are liver resident macrophages that localize within the lumen of the liver sinusoids and are the first immune cells in the liver that activate an important immune function against chronic inflammation^14^. The results of this activation include the production and secretion of a variety of inflammatory cytokines, reactive oxygen species, and growth regulatory mediators^14^. Although the function of KCs is still controversial in the carcinogenesis process, previous studies have reported that it has an important role in promoting the clonal expansion of preneoplastic cells required for DNA damage and cell growth regulation^8,14–17^. Our study also demonstrated that the number of KCs was increased during preneoplastic change in the experimental group. However, when there was neovascularization during carcinogenesis, normal sinusoids were gradually destroyed and the function of KCs might be changed^18^.

Sonazoid is a microbubble with negative charge which has a lipid shell, and these microbubbles are easily phagocytosed by KCs^19^. Recently published data had shown that the changes in KC function induced by cholangitis, could be successfully monitored by CEUS^20^. Therefore, the phagocytic function of KCs can be non-invasively monitored in the Kupffer-phase of CEUS, and the reduced contrast effect in the liver parenchyma has been attributed to KC dysfunction^10,11,21^. During non-invasive image monitoring, we found a significant decrease of liver parenchymal enhancement by Sonazoid in the experimental group after six weeks. Despite increased KC numbers, we speculate that Kupffer cells lose their functional ability to take up Sonazoid. Our hypothesis is that reduced phagocytic activity of the KCs is one of the features in the preneoplastic change during cholangiocarcinogenesis.

Moreover, in recent years it has become increasingly evident that hypoxia induced by the development of chronic inflammation causes cholestasis associated with a marked reduction in the expression of hepatobiliary transporters, and also contributes to carcinogenesis in chronic liver injury^9^. Hypoxia has a critical role in carcinogenesis, angiogenesis or tumor progression, and which is mediated by HIF-1α protein^22,23^. Therefore, it is important to investigate the feasibility of hypoxia monitoring and elucidate the involvement of HIF-1α expression during carcinogenesis.

Recent studies have highlighted the usefulness of PAI as a hypoxia monitoring tool in preclinical models^12,24,25^. PAI provides information on sO_2_ using the absorption characteristics of hemoglobin or deoxy-hemoglobin^26^. This method of measuring sO_2_ depends on the presence of an endogenous chromophore, ie, hemoglobin, in the target tissue^25^. Lee *et al*.^20^ reported PAI was useful for monitoring the serial change of liver sO_2_ in a mouse cholangitis model. Gerling *et al*.^24^ also reported the feasibility of measuring sO_2_ in a murine tumor model during tumor development and indicated that sO2 data were associated with hypoxic staining results. In our study, the sO_2_ of the experimental group was lower than that of the control group at four, six, and eight weeks, and it could be non-invasively monitored that hypoxia was induced in the preneoplastic change during cholangiocarcinogenesis. One hypothesis is that chronic liver injury could result in an increased resistance to blood flow and oxygen delivery, and thus leading to hypoxia during carcinogenesis and that it is also another feature in the preneoplastic change during cholangiocarcinogenesis. Yang *et al*.^27^ demonstrated that chronic liver injury by BDL transiently increased HIF-1α protein expression during cholestasis-associated carcinogenesis in a mouse model. Tian *et al*.^23^ also reported that overexpression of HIF-1α was emerging as an significant factor in carcinogenesis on the molecular level in CCA. It has been noted that HIF-1α transcriptionally activates VEGF protein, and which is a critical factor for angiogenesis, especially in areas of hypoxia^28^. Our study also demonstrated that the HIF-1α and VEGF expression increased in preneoplastic change during cholangiocarcinogenesis.

As a result of chronic inflammatory changes, biliary epithelium is known to be malignantly transformed through a multistep process with epithelial hyperplasia or dysplasia. Cholangiocarcinogenesis is a broad spectrum that leads to inflammatory changes and preneoplastic changes, and finally cancer development. The environmental changes described in our study was the changes of KCs and hypoxia-inducible factors although BilIN means a microscopic epithelial change. One hypothesis might be addressed regarding the tumor microenvironment in our results. CCA is characterized by a prominent desmoplastic stroma such as cancer-associated fibroblasts, tumor-associated macrophages and vascular cells. These factors undergoes profound changes in its composition and function during cholangiocarcinogenesis^29^. Therefore, our results could support the altered function of sinusoidal macrophages and dynamic changes of carcinogenesis-associated neovascularization. This study suggests that CEUS and PAI may be useful in monitoring carcinogenesis process to precancerous lesions even if cholangiocarcinogenesis is a broad spectrum that leads to inflammation, periductal fibrosis, precancerous dysplasia, and cancer development.

Our study has several limitations. First, the function of KC in our study was indirectly assessed by the Sonazoid phagocytosis ability of KC. However, KC is activated under pathological conditions and then functions in various ways through differentiation into M1 or M2 macrophages^14^. Previous animal studies have been performed only to demonstrate changes in phagocytic function of KCs in a rat model of nonalcoholic steatohepatitis^11,30^. Few studies have documented functional changes in KCs except for CEUS. In this study, we used an ultrasound contrast agent, which is easily phagocytosed by KCs. CEUS can assess KC phagocytic activity using the Kupffer-specific contrast agent^10,11^. The effect of ultrasound contrast agent phagocytosis by KCs can provide a stable, late-stage CEUS image called “Kupffer-phase” imaging^19^. A potential benefit of this CEUS imaging is that the quantitative changes in contrast enhancement values would provide data that reflects *in vivo* physiologic function of KC. Although we used the CEUS imaging that reflects the physiologic function of KC, further studies are needed to understand the implications of KC function changes during preneoplastic changes in liver parenchyma. In future studies, the phagocytosis by differentiated KCs during carcinogenesis needs to be directly evaluated through molecular work.

Second, some studies have used the pimonidazole injection model to assess the development of hypoxia during chronic liver injury or carcinogenesis^9,24,31^. However, we focused on non-invasive image monitoring of the hypoxic development in the carcinogenesis process. If pimonidazole can directly demonstrate the area of hypoxia, it could be direct evidence of hypoxia in the carcinogenesis process. On the other hand, there were a few studies that demonstrated the possibility of imaging monitoring in animal models using invasive needle or radioisotope in terms of hypoxia monitoring^32,33^. Recent interest in tumor hypoxic conditions has shifted to the area of diagnostic imaging due to the rapid development of diagnostic methods. PAI is a real-time, non-invasive and quantitative imaging method for hypoxia monitoring^12^. We were able to non-invasively monitor *in vivo* quantitative data of sO_2_ during preneoplastic changes in liver parenchyma through PAI without using radioisotope. PAI can provide quantitative information by measuring tissue sO_2_ using the optical absorption differences between oxygenated and deoxygenated hemoglobin^24,25^. However, the depth limitation of photoacoustic signals (ie, optical enabled penetration of 8 mm at excitation wavelength between 750 nm and 850 nm) still severely limits the clinical application of PAI^34^. Therefore, additional technical advances are needed to improve the detection and visibility of photoacoustic signals emitted from deeply located lesion.

Finally, our study could not establish the development of CCA arising from the chronic inflammation and NDMA administration. However, we found biliary epithelial hyperplasia with dysplastic cells, which meant the premalignant condition of CCA such as BilIN, in our animal model. Based on the degree of cellular and structural atypia, BilIN can be classified into three grades indicating low-grade dysplasia, high-grade dysplasia, and carcinoma *in situ* respectively^4^. The environmental changes described in our study was the changes of KCs and hypoxia-inducible factor, and our hypothesis might be addressed regarding the tumor microenvironment.

In conclusion, obstructive cholangitis and continued administration of NDMA promote preneoplastic changes during cholangiocarcinogenesis in mouse models. Our study demonstrated that KC dysfunction and hypoxic environmental changes were the causes of preneoplastic change and that we could non-invasively monitor these changes using CEUS with Sonazoid and PAI.

## Methods

This study was approved by our Institutional Animal Care and Use Committee (IACUC; No. 13-0369-C3A0) and was performed in accordance with the Guide for our IACUC and National Institute of Health Guide for the Care and Use of Laboratory Animals.

### Experimental Protocol

Six-week–old, male, BALB/c nude mice weighing 25 g were randomized into one of two groups as follows: (1) the experimental group (n = 33); and (2) the control group (n = 19). In the experimental group, NDMA was administered at a concentration of 12.5 ppm in drinking water for eight weeks, and only normal saline was administered in the control group.

Four weeks after the administration, BDL was performed in the experimental group, and the control group was made by sham operation. After intraperitoneal general anesthesia with 5 mg/kg zolazepam (Zoletil, Virbac, Carros, France) and xylazine hydrochloride (Rompun 2%, Bayer Korea, Seoul, Korea), the skin and peritoneum were incised along the midline of the animal. After lifting the entire liver, the common bile duct (CBD) before draining into the duodenum was gently isolated with moistened cotton gauze and microdissecting forceps. The distal CBD was ligated using 7-0 Prolene (Ethicon, Somerville, NJ) and the peritoneum was closed with Prolene.

CEUS and PAI were performed baseline, four-, six- and eight-weeks follow-up examinations following administration of NDMA or normal saline in order to observe bile duct dilatation, contrast agent uptake by KC, and changes in sO_2_ of liver parenchyma. Histopathologic examination was performed at six weeks (n = 28) and eight weeks (n = 24) **(**Fig. 1 **)**.

### Contrast-enhanced Ultrasound Examination

The CEUS examination was performed by a radiologist (J.H.K.) with 17 years of clinical experience performing liver ultrasound. The Aplio-500 ultrasound equipment (Toshiba Medical Systems Corp., Ototara, Japan) and a center frequency, 12-MHz linear transducer were used as the following parameters for B-mode imaging: a dynamic range of 65; a mechanical index of 0.9; a gain of 95; and a field-of-view depth of 1.5 cm. Ultrasonography for examining morphological features, including biliary dilatation, was performed in the B-mode.

After determining the optimal plane covering the wide range of liver parenchyma, ultrasound contrast agent Sonazoid (gaseous perflubutane; GE Healthcare, Waukesha, WI, USA) was intravenously injected at a dose of 3 μl/kg and was flushed with 0.2 mL saline. After 10 minutes of contrast agent injection, image acquisition was performed for 10 seconds and which corresponds to the Kupffer-phase in CEUS^19,35,36^. Shunichi *et al*.^36^ defined the Kupffer-phase images from the time point at which the liver parenchyma was enhanced starting 10 min after Sonazoid injection. Sontum *et al*.^35^ and Yanagisawa *et al*.^19^ had performed *in vitro* and *in vivo* experiments with Kupffer-phase imaging at 10 min after the injection of Sonazoid, when the hepatic parenchyma appeared homogeneously bright by physiochemical characteristics of Sonazoid. In addition, Miyata *et al*.^11^ defined the Kupffer-phase as 10 min after the injection of Sonazoid in a rat experiment, and Sugimoto *et al*.^37^ also observed the Kupffer-phase images in a similar method in a clinical study. The main advantage of Sonazoid is that it facilitates stable Kupffer-phase imaging with real-time visualization^38^.

CEUS images were acquired using a contrast harmonic image mode (CHI; Toshiba Medical Systems, Japan) with the following parameters: a transmission frequency of 12-MHz; a frame rate of 8 Hz; a dynamic range of 40; a mechanical index of 0.20; a gain of 73; and a depth of 1.5 cm. The evaluation of echogenicity by the contrast agent of all of the recorded raw data was performed using dedicated software (CHI-Q software, Toshiba Medical Systems, Japan). The average parenchymal echogenicity value was obtained by manually drawing the ROI three times while avoiding large blood vessels in the liver parenchyma. The serial change of liver parenchymal echogenicity in the Kupffer-phase imaging was measured at baseline, four-, six-, and eight-week follow-up examinations.

### High-resolution, Photoacoustic Ultrasound Examination

The examination of sO_2_ was performed by a radiologist (J.H.K.) with four years of PAI experience. A commercially available, high-resolution, photoacoustic imaging system (VisualSonics, Toronto, Ontario, Canada) and a central frequency, 21-MHz, LZ-250 PA linear transducer were used with the following parameters: a depth of 20.0 mm; a width of 23.04 mm; and a wavelength of 750/850 nm.

For the assessment of sO_2_, after placing the transducer at the target lobe of the liver, the liver was investigated by PAI in the OxyHemo-Mode so that the laser was fired with dual wavelengths of 750 and 850 nm during room air breathing^24^. As the binding of oxygen to hemoglobin alters its absorption spectrum according to different wavelengths, comparing the photoacoustic signal at 750 and 850 nm allowed for determination of the percent of oxygenation levels within the liver from the parametric maps of the sO_2_^24,25^.

In the sO_2_ parametric map of each target lobe, the ROI was drawn three times in order to obtain the average value. Serial change of liver sO_2_ in the PAI was measured at baseline and at the four-, six-, and eight-week follow-up examinations.

### Histologic Analysis

All of the mice were serially sacrificed at six weeks and eight weeks following administration of NDMA or normal saline. Among all of the mice (n = 52), there were 28 available specimens at six weeks and 24 specimens at eight weeks.

The specimens were fixed using 10% neutral-buffered formalin, embedded in paraffin, and cut into 4-um sections. Each section was stained with Harris’ hematoxylin solution and eosin Y (H&E) (Sigma, St. Louis, MO, USA) so that a pathologist (Y.Z.) could assess bile duct changes. Microscopic changes were evaluated according to the standard of human biliary pathology. Bile ductular reaction was defined as an increased number of bile ductules at the interface between portal tracts and the liver parenchyma. Biliary epithelial hyperplasia was micropapillary or pseudopapillary proliferation of non-dysplastic epithelial cells, while dysplasia was defined as the presence of atypical cellular changes including the irregular nuclear membrane, hyperchromasia, and loss of cellular polarity. The latter dysplastic changes corresponded to biliary intraepithelial neoplasia in human. The presence or absence of periductal inflammation and fibrosis was also examined. Histological evidence of stromal invasion was a requisite for the diagnosis of cholangiocarcinomas.

CD68 immunohistochemical staining was also performed using rabbit polyclonal antibody to CD68 (ab125212, 1:500 Abcam, Cambridge, UK) as the primary antibody and horseradish peroxidase (HRP)-labelled, polymer anti-rabbit antibody (K4003, Dako) as the secondary antibody.

CD68-stained images were analyzed using ImageJ 1.44 software (Wayne Rasband, NIH, MD, USA). Five hot spots (areas of higher CD68, positive brown staining compared with the rest of the tissue) were randomly selected at low magnification (×40) and the number of CD68-stained KCs was measured at high magnification (×200, 0.7386 mm^2^) in the hot spots. Finally, the fraction of the CD68-stained KCs from each slice was calculated five times and was then averaged.

### Western Blot Analysis

To verify the expression of HIF-1α and VEGF proteins, the total protein in the tissue samples was homogenized and lysed with ice-cold RIPA buffer (50 mmol/L Tris, 150 mmol/L NaCl, 1% NP40, protease, and phosphatase inhibitor). Protein extracts were separated using 10% polyacrylamide gel and were electro-transferred to nitrocellulose membranes. After blocking with 5% milk in TBST buffer (10 mmol/L Tris-HCl, 150 mmol/L NaCl, and 0.05% Tween-20), the membranes were probed with primary antibodies: HIF-1α (NB100-449, Novus Biologicals, Littleton, CO, USA); VEGF (ab46154, Abcam, CA, MA, USA); and actin (A2066, Sigma-Aldrich, St. Louis, MO, USA), followed by incubation with HRP-conjugated secondary antibody (T6778, Sigma-Aldrich, St. Louis, MO, USA). The target bands were visualized using enhanced chemiluminescence reagent (Amersham Biosciences), and the band images were quantified using ImageJ software (National Institutes of Health, Bethesda, MD, USA).

### Statistical Analysis

All statistical analyses were performed using SPSS version 21.0 (SPSS, Chicago, IL, USA). Results with a *P* value less than 0.05 were considered statistically significant. The parenchymal echogenicity and sO_2_ were assessed using the unpaired t-test to compare the significant differences between the experimental group and the control group. The unpaired t-test was also used to compare the CD68 count and the protein expression level (HIF-1α and VEGF) in each group.

## Electronic supplementary material

Supplementary Information
