# Supplementary material for: Imaging Monitoring of Kupffer Cell Function and Hepatic Oxygen Saturation in Preneoplastic Changes During Cholangiocarcinogenesis
Source: Sci Rep. 2017 Oct 27;7:14203. doi: 10.1038/s41598-017-14218-x (PMC5660185; doi:10.1038/s41598-017-14218-x)

**Imaging Monitoring of Kupffer Cell Function and Hepatic Oxygen Saturation in Preneoplastic Changes During Cholangiocarcinogenesis**

Seunghyun Lee^1^, Jung Hoon Kim^1,2*^, Jeong Hwa Lee^2^, Yoh Zen^3^, Joon Koo Han^1,2^

^1^Department of Radiology, Seoul National University Hospital

^2^Institute of Radiation Medicine, Seoul National University Hospital

^3^Department of Diagnostic Pathology, Kobe University Graduate School of Medicine

***Address correspondence to**: **Jung Hoon Kim, MD**

Department of Radiology, Seoul National University Hospital

101 Daehak-ro, Jongno-gu, Seoul, 110-744, Korea

Tel: 82-2-2072-2057, Fax: 82-2-743-6385

E-mail: jhkim2008@gmail.com

This research was partially supported by Basic Science Research Program through the National Research Foundation of Korea (NRF) funded by the Ministry of Education (No. 2013R1A1A2058033) and by a grant from the research was supported by Basic Science Research Program through the National Research Foundation of Korea(NRF) funded by the Ministry of  Science, ICT & Future Planning (2017R1A2B4004951).

**Figure S1. Full-length Western blots of HIF-1α, VEGF and actin. Regions of interests are highlighted in Fig. 4C.**


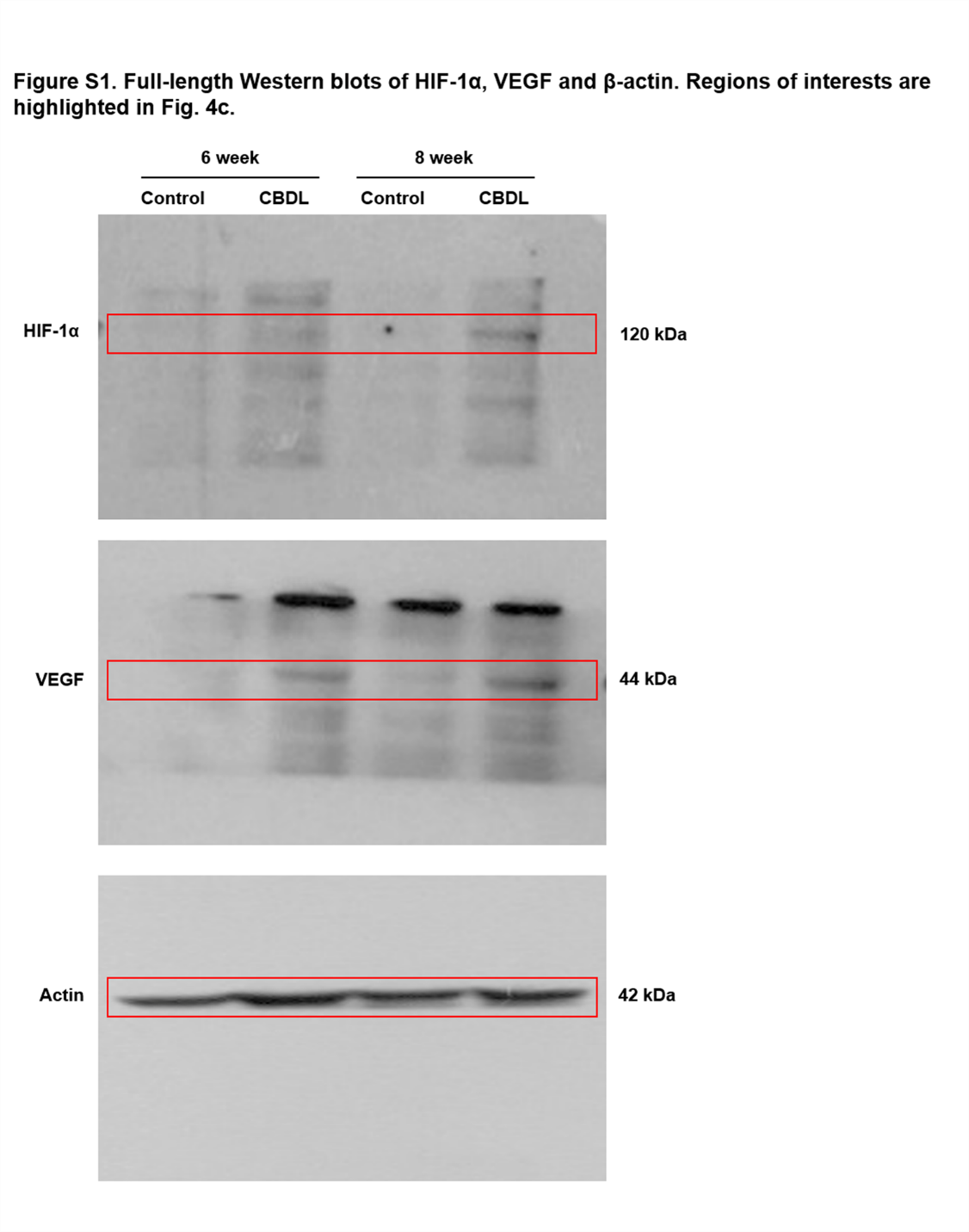

Supplement: Supplementary file 1 — Supplementary Information [file 41598_2017_14218_MOESM1_ESM.docx]
